# Case Conceptualization in Clinical Practice and Training

**DOI:** 10.32872/cpe.12103

**Published:** 2024-04-26

**Authors:** Eva Gilboa-Schechtman

**Affiliations:** 1 Department of Psychology and the Gonda Brain Research Center, Bar-Ilan University, Ramat-Gan, Israel; Department of Psychology, University of Trier, Trier, Germany

**Keywords:** case conceptualization, case formulation, integration, personalized treatment, supervision

## Abstract

Case conceptualization is central to the success of the therapeutic process. However, integrative case conceptualization research has lagged behind research on integrating therapeutic intervention techniques. A successful case conceptualization provides (a) a dynamic, context-sensitive, yet parsimonious model of the client’s functioning; (b) relevant treatment targets and associated assessment procedures; and (c) a treatment plan including intervention phases and potential obstacles. Success in case conceptualization is a core clinical competency goal for trainees in clinical psychology and a career-long learning goal even for expert clinicians. Emerging technological trends and the formation of adversarial collaborative teams may assist research on the utility of well-constructed case conceptualizations.

## Highlights

- Case conceptualization is central to the success of the therapeutic process.
- A successful case conceptualization promotes effective and personalized interventions.
- Research on integrative case conceptualization is lagging behind treatment by a factor of 1:100.

## Case Conceptualization: There Is Nothing More Practical Than a Good Theory

The global burdens associated with many common mental health conditions appear unaffected by diagnosis, prevention, and treatment advances, impacting individuals’, families’, and societies’ quality of life (Bruffaerts et al., 2018). Given this discouraging situation, calls are made to create trans-theoretical and empirically based ways of approaching mental health difficulties, combining elements from diverse treatment orientations to personalize treatments (Lutz & Schwartz, 2021; Schiepek & Pincus, 2023). In the following, I argue that the first step to such an integration is enhanced attention to a clinically central stage of the therapeutic process: case conceptualization.

Our lives are dynamic, and our goals, concerns, behaviors, and aspirations are modified in response to various challenges and opportunities. Our difficulties and strengths are similarly sensitive to various biological, psychological, and sociocultural processes and factors. Thus, there is wide agreement that psychiatric diagnoses are insufficient for clinical practice because they provide only a subset of the information clinicians need to help their patients. Case conceptualization, also known as case formulation, is created to weave a complete understanding of a person, of which diagnosis is only one aspect (McWilliams, 2021). Case conceptualization is a comprehensive and individualized understanding of a client’s presenting concerns, psychological symptoms, and interpersonal patterns. It thus integrates information from various sources, such as client interviews, assessment tools, and clinical observations. This information is used to formulate hypotheses regarding the factors responsible for developing (etiology) and maintaining the client’s pattern of difficulties. Case conceptualization aims to create a coherent and succinct narrative that provides a plausible framework for understanding the client’s difficulties and informs treatment planning (British Psychological Society, Division of Clinical Psychology, 2011; Eells, 2015, 2022a; Johnstone & Dallos, 2014; Persons, 2022; Sperry & Sperry, 2020).

Case conceptualization may serve as a linchpin between initial clinical assessment, personalized treatment development, and the intervention’s efficacy evaluation. A comprehensive, well-informed, theoretically rich (and thus, integrative), and source-diverse conceptualization will likely advance the construction of an effective and flexible treatment plan. In contrast, an insufficiently detailed or biased assessment is likely to result in a misguided conceptualization that leads, in turn, to an ill-formed treatment plan. Importantly, case conceptualization is an evolving process: throughout the therapeutic process, case conceptualization is *continually* refined and updated to reflect new information, insights, and responsivity to specific triggers and interventions.

*Integrative* case conceptualization – case conceptualization informed by multiple interwoven theoretical perspectives – is crucial if a treatment plan is also to be informed by such an integrative perspective. Yet, despite the centrality of case conceptualization for treatment planning, only a few dozen articles have been published in the last decade concerning case conceptualization (or formulation) and integration^1^1PSYCHNET for 2013-2023 identified 33 peer-reviewed articles published with “integrat*” and “case conceptualization” or “case formulation” as the keywords. This is compared to 3434 articles with “therap*” OR “treatment*,” OR “intervention*,” and “integrat*” in keywords.. This paucity is striking compared to a few thousand papers concerning treatment integration. This factor of 1:100 favoring the focus on intervention is maintained when we examine the direct comparison of two central research approaches – psychodynamic and cognitive-behavioral.^2^2PSYCHNET for 2013-2023 identified 65 articles published with the keywords “case conceptualization” or “case formulation” crossed with “*cognitive-behavioral” or CBT or “psychodynamic”*. This is compared to 9193 peer-reviewed articles with “therapy,” “treatment”, OR intervention,” were crossed with *cognitive-behavioral,” OR“psychodynamic.”*

## Case Conceptualization: An Attempt at Theoretical Integration

A case conceptualization needs to integrate diverse and complex information. *First*, it needs to include both nomothetic and idiographic information. The nomothetic information is derived from empirically supported models of individual differences, while the idiographic information contains specific data regarding the individual’s idiosyncratic history, concerns, motivations, and aspirations. One of the main goals of case conceptualization is to connect the specific patterns of distress that bring the individual to treatment (idiographic concerns) with this rich database of nomothetic information. For example, it is important to assess which symptoms of distress are specifically significant for this client. Indeed, recent research suggests the need to broaden and refine our definitions of distress even in the most well-known conditions, such as depression, post-traumatic stress disorder, and social anxiety (Gilboa-Schechtman, 2020; Gilboa-Schechtman et al., 2020; Keshet & Gilboa-Schechtman, 2017). Moreover, standard clinical measures of depression have been criticized for measuring domains of limited relevance to patients and leaving out significant areas of concern, such as sick leave, work difficulties, or impaired relationships (Fried & Nesse, 2015). *Second*, a case conceptualization needs to integrate information from several time frames. A macro timescale (decades, years) may include information concerning the client’s personal history from early childhood to the present and their aspirations and concerns about the future. The ability of the client to achieve developmental milestones (such as moving to independent living) and handle common stressors (loss of a relationship or a job) is important for the eventual understanding of the person’s vulnerabilities and areas of resilience. A meso timescale (weeks, days) may include data concerning their affect, behavior, cognition, and physiological reactions in the period preceding their turn to treatment. Finally, micro timescale (hours, minutes) information may include multi-modal data concerning their response to in-session interactions. This information may be used to examine the way clients experience their therapists and therapists’ responses to their clients. *Third*, case conceptualization needs to consider how culture (communal, individualistic) and societal context (such as social class or sexual orientation) impact our lives. Importantly, culture affects not only our values (e.g., honoring the dead, obedience), beliefs (say, “direct communication is important,” “time progresses linearly”), and coping strategies (such as help-seeking from family and friends, prayers, and spiritual practices), but also the emotions that we value and cherish (for example, the value of individualistic pride appears to be higher in Western than non-Western cultures, Kitayama et al., 2006). Social class impacts thoughts, feelings, and behavior (Manstead, 2018; Stephens et al., 2014). Understanding how our identities, shaped by culture and context, intersect offers a greater depth of the client’s history and challenges. *Fourth*, the construction of a comprehensive case conceptualization may involve, when possible, information from several perspectives, including the individual’s own experiences, as well as input from family members, other healthcare providers, and the therapist’s own evaluation of the client’s behavior during the assessment and the treatment processes.

Clinically, the development of a case conceptualization involves several steps. *First*, identifying the client’s presenting problems, such as the specific symptoms, issues, or difficulties the client is experiencing. These can be emotional, cognitive, behavioral, or interpersonal in nature. *Second*, understanding the client’s background and context by exploring their personal history, family dynamics, social environment, and cultural background may contribute to developing or maintaining their difficulties. Indeed, the first two stages offer the opportunity to utilize diverse theoretical orientations, as different orientations emphasize diverse sources of information as significant (e.g., early family dynamics, genetic factors, learning history). *Third*, assessing the client’s strengths and resources: social and intellectual skills, coping mechanisms, and social support. *Fourth*, formulating hypotheses about the underlying mechanisms or patterns that contributing to the client’s difficulties. Crucially, these hypotheses can be informed by various psychological theories or models, such as cognitive-behavioral (CBT), psychodynamic, humanistic, or trans-theoretical perspectives (e.g., Eubanks & Goldfried, 2019). CBT makes an important distinction between etiological and maintenance factors for disorders. Including diverse factors in the conceptualization (Wong & Rapee, 2016 in the case of social anxiety disorder) may clarify the immediate and long-term treatment targets. *Fifth*, establishing treatment goals. In collaboration with the client, the clinician can set specific, measurable, and achievable goals for therapy, which will address the identified problems and promote overall well-being. Again, such goals may be enriched by inputs from trans-theoretical models of psychotherapy (Bailey & Ogles, 2023) and include, besides, reduction of distress, increase in insight, and in self-efficacy. *Finally*, developing an individually tailored intervention plan that outlines the therapeutic approaches, techniques, and strategies that will be employed to help the client achieve their goals. Thus, case conceptualization serves as a roadmap for both the therapist and the client, guiding the direction and focus of therapy and helping to monitor progress and outcomes.

## Training Implications

Case conceptualization is a widely agreed upon core clinical competency (Eells, 2022a; Page et al., 2008; Rief, 2021; Sperry & Sperry, 2020). This competency is based on theories of personality and psychopathology, coursework on assessment and diagnostics, and the treatment outcome literature learned in lectures and dialogues conducted during clinical supervision. Thus, clinical supervision aims to assist supervisees in shifting from *abstract knowledge about* case conceptualization to the *case-specific clinical implementation* of this knowledge (Page et al., 2008).

Case conceptualization can be thought of as a model of the client’s intra- and interpersonal dynamics. Given that models are inherently “wrong” in that they are incomplete approximations of reality, the utility of a model for clinical inference is determined by its ability to provide *actionable insights* for psychotherapy (Fried, 2020). Clinical supervision needs to help trainees find a compromise between simple models and elaborated models by emphasizing that the model is as good as the insights into treatment planning it allows. Whereas most beginner clinicians can identify some presenting problems, strengths, and precipitating factors, elements of the conceptualization concerning etiological and maintaining mechanisms are typically more difficult to articulate. Formulating and testing nuanced hypotheses inherent in each conceptualization is an elusive yet important part of the conceptualization (Ridley et al., 2017). This elusiveness is illustrated in the study by Eells and colleagues, who found that experienced clinicians with decades of professional experience were almost as likely to include a psychological mechanism in their case conceptualization as novices (Eells et al., 2005). Another study with experienced clinicians providing a psychodynamic conceptualization found that many clinicians used a relatively low inference level and an experience-near terminology, again suggesting that many therapists introduce few maintenance mechanisms in their case conceptualizations (Sørbye et al., 2019). Providing a specific structure for the case conceptualization and encouraging trainees to refer to all components of the conceptualization may improve the completeness and quality of their models.

One of the most important tasks of the supervisor is the gentle yet consistent encouragement to construct a “good enough” conceptualization at the *onset* of treatment. There is extensive agreement that an *early* attempt to construct a case conceptualization is an important and necessary foundation for competent practice in several approaches (CBT; Kuyken et al., 2009; Persons, 2008), dynamic therapy (McWilliams, 2011; Shedler, 2022), and interpersonal therapy (Hopwood et al., 2019). Moreover, there is an emergent agreement regarding the clinical importance of the involvement of clients in case conceptualization, goal setting, and treatment planning (Beck et al., 1979; Hopwood et al., 2019; Kuyken et al., 2009; McFarquhar et al., 2023; Tee & Kazantzis, 2011). Such client involvement is crucial for enhancing the transparency of clinical practice and facilitating the client’s understanding of – and, therefore, engagement in – therapy itself.

A competently developed, high-quality conceptualization goes beyond a summary of information about the client (Eells et al., 2005). It is important to enhance the trainees’ ability to check their conceptualization for completeness (Eells, 2013). Specifically, the case conceptualization should be (a) *comprehensive* in addressing multiple aspects of a client’s functioning; (b) *understandable* to the client and thus use language that is precise and non-technical; (c) *parsimonious* yet not simplistic; (d) *coherent*, providing an internally consistent model of the individual’s problems, explaining the presenting complaints by reference to predisposing vulnerabilities and strengths, precipitating events, etiological and maintaining factors; (e) *science-informed,* offering explanatory hypotheses linked to knowledge about personality and psychopathology; (f) *generative*, highlighting the ways in which the treatment plan logically flows from the explanatory hypotheses and predicts measurable outcomes; and finally, (g) *cohesive*, offering a treatment plan that links the hypotheses with a therapeutic course of action.

Ultimately, it is important to stress to trainees that the skill of conceptualizing involves career-long learning. Case conceptualization is a complex process that requires clinicians to draw on a wide range of theoretical and practical knowledge to understand each client’s unique needs and challenges. By promoting career-long learning, we can continually expand our knowledge base and develop new skills that can enhance our ability to formulate effective treatment plans for our clients. Additionally, ongoing mentoring and supervision can provide clinicians with feedback and guidance that can help us refine our case formulation skills and approaches. Supervisors who view clinical science as a process of continuous development and who model intellectual humility and healthy skepticism as parts of their professional improvement process are likely to foster up-to-date psychological methods among their supervisees. This, in turn, can lead to more effective treatment outcomes for clients and enhance the overall quality of clinical practice.

## Research on Case Conceptualization

Research on case conceptualization traditionally examined questions of validity and reliability (Easden & Kazantzis, 2018; Eells, 2022b). Most studies evaluate the *reliability* of various case conceptualization methods to assess whether different clinicians, using the same case conceptualization approach, arrive at similar or consistent conceptualizations for a given client or case. This line of research typically involves comparing the conceptualizations of multiple clinicians who independently review the same case information (Easden & Kazantzis, 2018; Persons & Hong, 2015).

The *validity* of case conceptualization examines the extent to which different conceptualization methods accurately capture and explain the client’s presenting problems, underlying mechanisms, and treatment progress (Easden & Kazantzis, 2018; Horowitz et al., 1995). Validity is typically established when predictions made by a case conceptualization match actual treatment outcome or when different approaches arrive at similar conclusions (Bucci, French, & Berry, 2016; Mumma, 2011; Mumma et al., 2018). Indeed, examining the clinical advantage of case conceptualization involves assessing whether more accurate, thorough, or complete case conceptualizations are associated with better treatment outcomes, such as reduced distress, increased client satisfaction, or enhanced therapeutic alliance, yet only scant research has examined this question (Bucci et al., 2016). To further examine the effectiveness of different case conceptualization approaches, researchers may conduct well-powered randomized controlled trials or comparative studies investigating whether certain case conceptualization approaches lead to better treatment outcomes than others or whether specific approaches are more suitable for particular client populations, problem areas, or therapeutic modalities (see Eells, 2022b for a review of initial attempts in this direction).

Additional important questions involve the role of therapist factors (e.g., training, experience, theoretical orientation) in the comprehensiveness and effectiveness of case conceptualization. Finally, the importance of the timing of case conceptualization may be explored, and the timing of the construction and sharing of case conceptualization with clients may be examined. For example, research may compare sharing a case conceptualization with the client early in treatment (in the first third of the treatment sequence) versus late treatment (in the middle or the final third of the treatment sequence). Such research can use case conceptualization methodologies to translate the idiographic nature of psychotherapy into quantitative research designs (Haynes et al., 2009; Kramer, 2020). For example, when idiosyncratic mechanisms are defined (e.g., over-utilization of cues of social status as opposed to cues of affiliation in social anxiety, Gilboa-Schechtman, 2020), the outcomes of these specific mechanisms can be assessed in a quantitative design.

## A Way Forward

An ongoing challenge for reliable, valid, and therapeutically useful clinical case conceptualization is the time constraints for completing this complex task. However, we are witnessing exciting advances promising to assist us in the timely completion of this task. On the methodological side, with the advancement of ecological momentary assessment (EMA, mostly relying on self-report), ambulatory monitoring (which may include physiological data), and routine outcome assessment (e.g., Schaffrath et al., 2022) we can look forward to collecting a wealth of data about a single individual from a variety of intra-personal (including physiology, behavior, passive sensing of a digital footprint, and expressive signals such as voice, as well as subjective self-report) as well as interpersonal (e.g., family, close friends) sources. With advancing technology, techniques enabling the automated analysis of such intensive data will become increasingly available. Such innovations require a willingness to critically evaluate and adapt one’s own clinical practice based on emerging clinical tools, empirical findings, and client feedback.

On the theoretical side, a unified effort to foster adversarial collaborations between representatives of diverse schools of clinical thought in creating a comprehensive scheme for case conceptualization appears to be needed. These collaborations may enhance trans-diagnostic approaches and pluralism by increasing awareness of confirmation biases inherent in any one approach (Doherty et al., 2019). Adversarial collaborations may also help representatives from various schools of thought clarify and refine the assumptions underlying the components needed for a successful case conceptualization. Such collaborations may strengthen the link between research in psychopathology and psychotherapy and foster integration between treatment orientations.
